# Dual Angiotensin Receptor and Neprilysin Inhibitor Ameliorates Portal Hypertension in Portal Hypertensive Rats

**DOI:** 10.3390/pharmaceutics12040320

**Published:** 2020-04-02

**Authors:** Shao-Jung Hsu, Hui-Chun Huang, Chiao-Lin Chuang, Ching-Chih Chang, Ming-Chih Hou, Fa-Yauh Lee, Shou-Dong Lee

**Affiliations:** 1 Division of Gastroenterology and Hepatology, Department of Medicine, Taipei Veterans General Hospital, Taipei 11217, Taiwan; sjhsu@vghtpe.gov.tw (S.-J.H.); hchuang2@vghtpe.gov.tw (H.-C.H.); mchou@vghtpe.gov.tw (M.-C.H.); fylee@vghtpe.gov.tw (F.-Y.L.); sdlee@vghtpe.gov.tw (S.-D.L.); 2 Faculty of Medicine, National Yang-Ming University School of Medicine, Taipei 11217, Taiwan; clchuang@vghtpe.gov.tw; 3 Division of General Medicine, Department of Medicine, Taipei Veterans General Hospital, Taipei 11217, Taiwan

**Keywords:** angiotensin receptor neprilysin inhibitor, natriuretic peptide, portal hypertension, renin-angiotensin-aldosterone system

## Abstract

Background: Portal hypertension is characterized by exaggerated activation of the renin-angiotensin-aldosterone axis. Natriuretic peptide system plays a counter-regulatory role, which is modulated by neprilysin. LCZ696 (sacubitril/valsartan) is a dual angiotensin receptor and neprilysin inhibitor. This study evaluated the effect of LCZ696 on portal hypertensive rats. Methods: Portal hypertension was induced by partial portal vein ligation (PVL) in rats. LCZ696, valsartan (angiotensin receptor blocker), or normal saline (control) was administered in PVL rats for 10 days. Then, hemodynamic and biochemistry data were obtained. The hepatic histology and protein expressions were surveyed. On the parallel groups, the portal-systemic shunting degrees were determined. Results: LCZ696 and valsartan reduced mean arterial pressure and systemic vascular resistance. LCZ696, but not valsartan, reduced portal pressure in portal hypertensive rats (control vs. valsartan vs. LCZ696: 15.4 ± 1.6 vs. 14.0 ± 2.3 vs. 12.0 ± 2.0 mmHg, control vs. LCZ696: *P* < 0.05). LCZ696 and valsartan improved liver biochemistry data and reduced intrahepatic Cluster of Differentiation 68 (CD68)-stained macrophages infiltration. Hepatic endothelin-1 (ET-1) protein expression was downregulated by LCZ696. The portal-systemic shunting was not affected by LCZ696 and valsartan. Conclusion: LCZ696 and valsartan reduced mean arterial pressure through peripheral vasodilation. Furthermore, LCZ696 significantly reduced portal pressure in PVL rats via hepatic ET-1 downregulation.

## 1. Introduction

In chronic liver diseases, an increase of intrahepatic resistance develops due to chronic inflammation and fibrosis, which impedes hepatic blood flow then induces portal hypertension [1]. At the same time, peripheral vasodilation takes place, followed by renin-angiotensin-aldosterone system (RAAS) activation and sodium and fluid retention, with the initial aim to compensate for inadequate intravascular effective volume [2]. The RAAS regulates vascular tone and affects systemic and portal hemodynamics through mainly angiotensin II and aldosterone. However, exaggerated RAAS activation in portal hypertension leads to many dreadful complications such as ascites’ formation and hepatorenal syndrome, which has raised the therapeutic application of RAAS inhibitors, such as angiotensin converting enzyme inhibitor, angiotensin receptor blocker (ARB), and direct renin inhibitor [3,4]. Unfortunately, the therapeutic efficacy of these agents is limited because they cannot ideally inhibit the activity of RAAS [5]. On the other hand, another counter-regulatory pathway, the so-called natriuretic peptide (NP) system, is also critical in maintaining hemodynamic homeostasis [6]. The NP system consists of atrial NP (ANP), B-type NP (BNP), and C-type NP (CNP). NPs activate transmembrane receptors to upregulate guanylate cyclase and to increase levels of the second messenger cyclic guanosine monophosphate and its effector, protein kinase G. Via these actions, NPs counteract the RAAS and elicit vasodilation and diuresis.

A key modulator of the NP system is neprilysin (neutral endopeptidase 24.11) which catalyzes ANP, BNP, and CNP as well as the degradation of endothelin-1 (ET-1) and angiotensin II [7]. Inhibition of neprilysin can enhance the effect of the NP system. However, neprilysin inhibition results in activation of the RAAS. Therefore, in order to be clinically effective, neprilysin inhibitor requires concomitant inhibition of RAAS. LCZ696 (sacubitril/valsartan, Entresto TM, Novartis, C_24_H_29_N_5_O_3_.C_24_H_29_NO_5_.5/2H_2_O.3Na, molecular weight: 915.98) is a dual angiotensin II receptor and neprilysin inhibitor (ARNI), which has been documented to be beneficial in patients with chronic heart failure with reduced ejection fraction [8]. LCZ696 is composed of two molecular moieties (in a 1:1 molar ratio) in a single crystalline complex of valsartan (a kind of ARB) and sacubitril (neprilysin inhibitor) [9]. In addition, emerging data show that LCZ696 is safe and well tolerated in patients with mild to moderate hepatic impairment [10].

In portal hypertension, an increase of intrahepatic vascular response to vasoconstrictor and enhancement of intrahepatic vascular resistance play important roles in the pathophysiology [11]. Sansoè et al. showed that neutral endopeptidase inhibition by candoxatrilat reduced portal pressure and intrahepatic vascular resistance in cirrhotic rats, suggesting the beneficial effect by elevating NPs to ameliorate portal hypertension [12]. Another study showed that selective activation of ANP receptor induced splanchnic vasoconstriction and decreased portal pressure in cirrhotic rats, also implicating the therapeutic effect of NPs in portal hypertension [13].

Therefore, ARNI might have therapeutic potential in portal hypertensive state. This study thus aimed to examine its influences on the hemodynamic response, portal-systemic collateral formation, liver and renal function, hepatic protein expressions, and histological changes in portal hypertensive rats.

## 2. Materials and Methods

### 2.1. Animal Model 

Male Sprague-Dawley rats (300–350 g) were caged at 24 °C with a 12-h light-dark cycle and free access to food and water until the time of experiments. Portal hypertension was induced by partial portal vein ligation (PVL). Sham operation was conducted as the surgical control. Survival surgery and hemodynamic study were performed under Zoletil (50 mg/kg, intramuscular injection) anesthesia. Guide for the Care and Use of Laboratory Animals (The National Academies Press, National Institutes of Health, USA, publication, 8th Ed., revised 2011) was followed. This study was approved by Taipei Veterans General Hospital Animal Committee (IACUC 2018-052).

### 2.2. Experimental Design

After PVL or sham operation, rats were randomly allocated into 3 groups to receive oral gavage of LCZ696 (50 mg/kg), valsartan (25 mg/kg), or placebo (normal saline 0.2 mL, vehicle control group) for 10 days. On the 10th day, body weight (BW) and hemodynamic data, including mean arterial pressure (MAP), heart rate (HR), portal venous pressure (PP), superior mesenteric arterial blood flow (SMAf), superior mesenteric arterial resistance (SMAR), portal venous blood flow (PVf), cardiac index (CI), and systemic vascular resistance (SVR). were measured. Blood was withdrawn for measuring plasma levels of creatinine, alanine aminotransferase (ALT), aspartate aminotransferase (AST), and total bilirubin at the end of experiments. Livers were dissected for protein expression analyses. The hepatic histopathological changes and immunohistochemical staining were conducted. In another 3 parallel PVL groups, the impact of LCZ696, valsartan, or vehicle on portal-systemic shunting degree was determined. 

### 2.3. Measurement of Systemic and Portal Hemodynamics

The right femoral artery and mesenteric vein were cannulated with polyethylene (PE)-50 catheters connected to a Spectramed DTX transducer (Spectramed Inc., Oxnard, CA, USA). The external zero reference was placed at the level of the midportion of the rat. Continuous recordings of MAP, HR, and PP were performed on a multichannel recorder (model RS 3400, Gould Inc., Cupertino, CA, USA). Cardiac output was measured by thermodilution, as previously described [14]. Briefly, a thermistor was placed in the aortic arch just distal to the aortic valve and the thermal indicator (100 μL of normal saline) was injected into the right atrium through a PE-50 catheter. The aortic thermistor was connected to a Columbus Instruments Cardiotherm 500-AC-R (Columbus Instruments International Co., Columbus, OH, USA). Five thermodilution curves were obtained for each cardiac output measurement. The final cardiac output value was obtained from the arithmetic mean. CI (mL/min/100 g BW) was calculated as cardiac output per 100 g BW of rat. SVR (mmHg/mL/min/100 g BW) was calculated by dividing MAP by CI. 

### 2.4. Measurement of Portal Venous and Superior Mesentery Arterial Hemodynamics

The measurements of PVf (mL/min/100 g BW) and SMAf (mL/min/100 g BW) were performed as previously described [15]. In brief, after carefully dissecting the portal vein and superior mesentery artery from their surrounding soft tissue, PVf and SMAf were measured using a nonconstrictive perivascular ultrasonic transit-time flow probe (l RB, 1-mm diameter, Transonic Systems, Ithaca, NY, USA). SMAR (mmHg/mL/min/100 g BW) was calculated by dividing (MAP minus PP) by SMAf.

### 2.5. Portal-Systemic Shunting Analysis

The portal-systemic shunting degree was determined using the color microsphere technique as described previously [16]. In brief, 30,000 15-μm color microspheres (Dye Track, Triton Technology, San Diego, CA, USA) were slowly injected into the spleen. In “normal” condition, the microspheres would get into the liver through the splenic vein, being trapped in the liver. However, in portal hypertension, most of the microspheres bypass the stiff liver and escape through portal-systemic shunts into the lung where they become trapped. After the microspheres were injected into the spleen, the rats were euthanized and the livers and lungs were dissected and placed in new polypropylene centrifuge tubes. The numbers of microspheres were determined following the protocol provided by the manufacturer. The degree of portal-systemic shunting was calculated as the number of microspheres in the lung divided by the sum of microspheres in the liver and lung. Assuming a worst-case scenario in which two-thirds of the microspheres were trapped in the spleen, this technique could still detect a minimum shunt of 3.5%.

### 2.6. Histopathological and Immunohistochemical Staining of Liver

The liver was dissected free and fixed in 10% formalin solution. The sections were stained with hematoxylin and eosin (H&E) and examined by light microscopy. The immunohistochemical staining study was performed with anti-Cluster of Differentiation 68 (CD68) antibody (diluted 1:200, ab31630, Abcam, Cambridge, UK) to detect intrahepatic CD68-positive stained macrophages, which could indicate the severity of intrahepatic inflammation. The numbers of CD68-positive cells per high-power field (magnification 200×) were counted by a semiquantification method [17].

### 2.7. Western Blot Analysis for Various Protein Expressions

Liver tissue was immediately frozen in liquid nitrogen and stored at −80 °C until required. The protein extracts were made by pulverization in a grinder with liquid nitrogen, using a ratio of 1 mL of lysis buffer (phosphate-buffered solution containing 1% Nonidet P-40, 0.5% sodium deoxycholate, 0.1% sodium dodecyl sulfate (SDS) and 0.05% protease inhibitor cocktail solution (Roche Diagnostics GmbH, Penzberg, Germany) for each 100-mg powdered liver sample. Blots were incubated with the primary antibody (anti-cyclooxygenase (COX)-1 receptor, -COX-2 receptor (1:500, Santa Cruz Biotechnology, Santa Cruz, CA, USA), anti-endothelin-1 (ET-1) (1:1000; Cell Signaling Technology, Danvers, Massachusetts, USA), anti-vascular endothelial growth factor (VEGF) (1:1000, Santa Cruz Biotechnology), anti-endothelial nitric oxide synthase (eNOS), anti-inducible nitric oxide synthase (iNOS) (1:1000, Millipore Corporation, Billerica, MA, USA), antiphosphorylated eNOS (1:1000, Cell Signaling Technology), antiphosphorylated iNOS (1:1000; Abcam plc, Cambridge, UK), anti-nuclear factor kappa B (NFκB) p65 antibody (anti-NFκB p65) (1:200, Santa Cruz Biotechnology), antiphosphorylated-NFκB p65 (1:1000, Abcam plc), antinuclear factor of kappa light polypeptide gene enhancer in B-cells inhibitor alpha (IκBα) (1:10,000, Abcam plc); antiphosphorylated IκBα (1:1000, Cell Signaling Technology)) for 90 min at room temperature and washed. Then the blots were incubated for 90 min with the secondary antibody (horseradish peroxidase-conjugated goat anti-mouse immunoglobulin G antibody, diluted with 3% nonfat dry milk in Tris-Buffered Saline, 0.1% Tween®, Merck KGaA, Darmstadt, Germany) and washed. Subsequent detection of the specific proteins were performed by enhanced chemiluminescence (5-bromo, 4-chloro, 3-indolylphosphate /Nitro-Blue Tetrazolium substrate solution, Amresco Co., Solon, OH, USA). The β-actin (Chemicon, Merck-Millipore, Burlington, Massachusetts, USA) was used as internal control. With a computer-assisted video densitometer and digitalized software (Kodak Digital Science^TM^ ID Image Analysis Software, Eastman Kodak Co., Rochester, NY, USA), the blots were scanned and photographed then the signal intensity (integral volume) of the appropriate bands was analyzed. 

### 2.8. Drugs

LCZ696 and valsartan were purchased from Novartis, Taiwan. LCZ696 and valsartan were dissolved in normal saline. All solutions were freshly prepared on the days of experiments.

### 2.9. Data Analysis

The results are expressed as mean ± standard deviation. Statistical analyses were performed by one-way ANOVA test with post hoc Tukey analysis and the survival curve analysis using log-rank test. Results were considered statistically significant at a two-tailed *P* value less than 0.05.

## 3. Results

### 3.1. Mortality Rates of Control, Valsartan- and LCZ696-Treated PVL, and Sham-Operated Rats

All rats survived throughout the treatment period with LCZ696, valsartan, or vehicle.

### 3.2. Hemodynamic Changes of Sham-Operated and PVL Rats after Valsartan and LCZ696 Treatments 

Table 1 displays the hemodynamic changes of vehicle- (control, *n* = 6), valsartan- (*n* = 5), and LCZ696-treated (*n* = 5) sham-operated rats. Valsartan and LCZ696 significantly reduced mean arterial pressure (control vs. valsartan vs. LCZ696: 145 ± 11 vs. 121 ± 19 vs. 104 ± 15 mmHg, valsartan and LCZ696 vs. control: *P* < 0.05). LCZ696 significantly reduced systemic vascular resistance and valsartan tended to reduce systemic vascular resistance but the statistical significance was not reached (control vs. valsartan vs. LCZ696: 3.9 ± 0.4 vs. 3.2 ± 0.6 vs. 2.7 ± 0.5 mmHg/mL/min/100 g, LCZ696 vs. control: *P* < 0.05; valsartan vs control: *P* = 0.054). The body weight, portal pressure, heart rate, portal venous flow, superior mesentery arterial flow, superior mesentery arterial resistance and cardiac index were not significantly different among the control, and valsartan- and LCZ696-treated groups.

Table 2 shows the hemodynamic changes after different treatments in partial portal vein ligation (PVL)-induced portal hypertensive rats. Valsartan and LCZ696 significantly reduced mean arterial pressure (MAP) as well as systemic vascular resistance (SVR) (control vs. valsartan vs. LCZ696: MAP = 126 ± 5 vs. 103 ± 16 vs. 90 ± 12 mmHg, valsartan and LCZ696 vs. control: *P* < 0.05; SVR = 3.9 ± 0.4 vs. 2.7 ± 1.1 vs. 2.3 ± 0.5 mmHg/mL/min/100 g, valsartan and LCZ696 vs. control: *P* < 0.05). LCZ696 also reduced portal pressure (PP) and superior mesentery arterial resistance (SMAR) (control vs. valsartan vs. LCZ696: PP = 15.4 ± 1.6 vs. 14.0 ± 2.3 vs. 12.0 ± 2.0 mmHg, LCZ696 vs. control: *P* < 0.05; SMAR = 18.6 ± 1.8 vs. 12.9 ± 5.7 vs. 11.3 ± 3.6 mmHg/mL/min/100 g; LCZ696 vs. control: *P* < 0.05). The body weight, heart rate, portal venous flow, superior mesentery arterial flow and cardiac index were not significantly different among these 3 groups.

### 3.3. Effects of Valsartan and LCZ696 Treatment on Biochemistry and Histopathological Changes of Liver in PVL Rats

**Figure 1:**
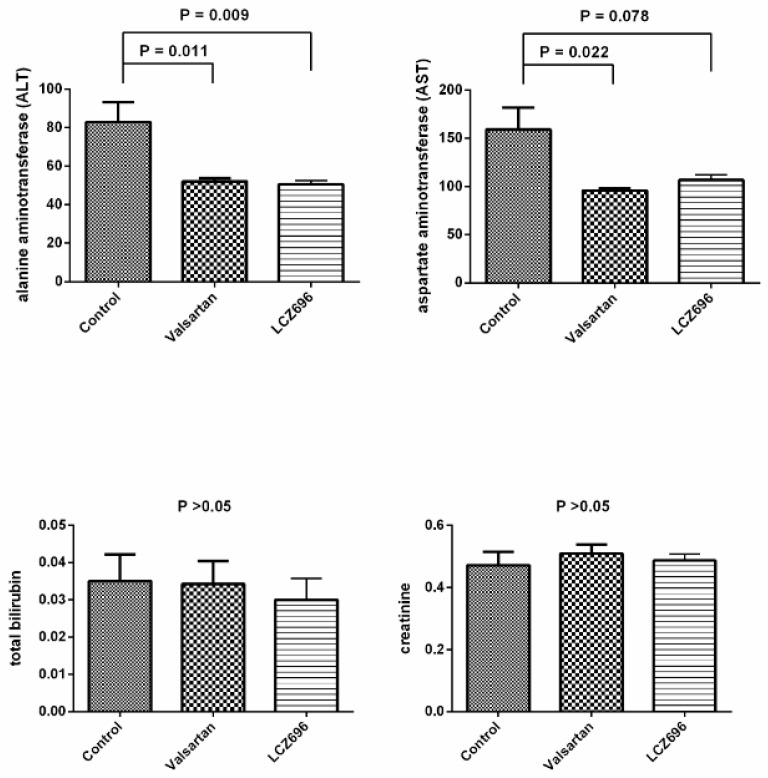
Biochemical data of partial portal vein ligation (PVL) rats treated by vehicle (control), valsartan, or LCZ696 (sacubitril/valsartan). Valsartan and LCZ696 significantly decreased the plasma levels of alanine aminotransferase (ALT) (both *P* < 0.05). In addition, valsartan decreased the plasma level of aspartate aminotransferase (AST) (*P* < 0.05). The total bilirubin and creatinine levels were not significantly affected by valsartan and LCZ696.

Figure 1 depicts the biochemistry data of PVL rats after vehicle (control, *n* = 5), valsartan (*n* = 7), or LCZ696 (*n* = 7) treatment. Valsartan and LCZ696 significantly decreased ALT levels (control vs. valsartan vs. LCZ696: ALT = 83 ± 25 vs. 52 ± 4 vs. 50 ± 5 IU/L, valsartan and LCZ696 vs. control: *P* < 0.05). Valsartan significantly decreased the plasma levels of AST, and LCZ696 tended to reduce the AST level but the statistical significance was not reached (AST = 159 ± 56 vs. 96 ± 6 vs. 109 ± 14 IU/L; valsartan vs. control, *P* < 0.05; LCZ696 vs. control, *P* = 0.078). The plasma levels of total bilirubin and creatinine were not significantly different among these 3 groups.

**Figure 2:**
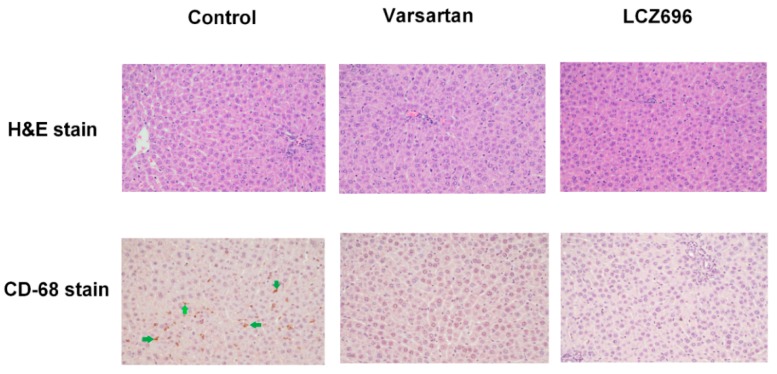
Liver histology and immunochemical staining of PVL rats treated by vehicle (control), valsartan, or LCZ696. The representative hematoxylin and eosin staining images show similar architecture of liver tissue among these 3 groups (magnification 200×, upper panel). Many Cluster of Differentiation 68 (CD68)-positive staining cells (the representative brown cell with irregular shape as indicated by green arrow) were noted in the control group. In contrast, they were nearly absent in those treated by valsartan and LCZ696 (magnification 200×, lower panel).

The hepatic H&E staining of PVL rats showed normal architecture of hepatic tissue after vehicle, valsartan, and LCZ696 treatments (upper panel of Figure 2). However, there were prominent CD68-positive stained cell infiltrations in the liver of PVL rats, which were significantly attenuated by valsartan and LCZ696 (lower panel of Figure 2).

### 3.4. Portal-Systemic Shunting in the Control (n = 6) and Valsartan- (n = 6) and LCZ696-Treated (n = 6) PVL Rats

**Figure 3:**
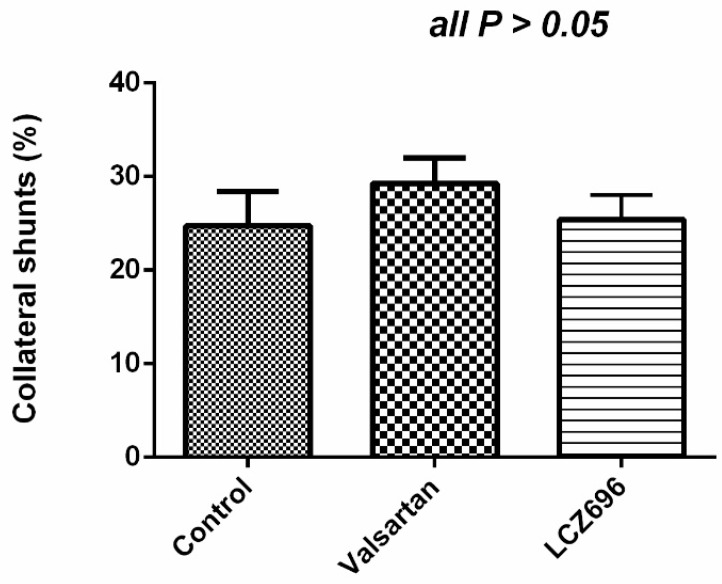
Portal-systemic shunting degree of PVL rats treated with vehicle, valsartan, or LCZ696. Valsartan and LCZ696 did not significantly affect the shunting degree (*P* > 0.05).

Valsartan and LCZ696 did not influence the degree of portal-systemic collateral shunting in the PVL rats (control vs. valsartan vs. LCZ696: 24.7 ± 0.1 vs. 29.3 ± 0.1 vs. 25.4 ± 0.1, *P* > 0.05; Figure 3).

### 3.5. Protein Expressions in the Liver of PVL Rats Treated by Vehicle (n = 6), Valsartan (n = 8), or LCZ696 (n = 8)

**Figure 4:**
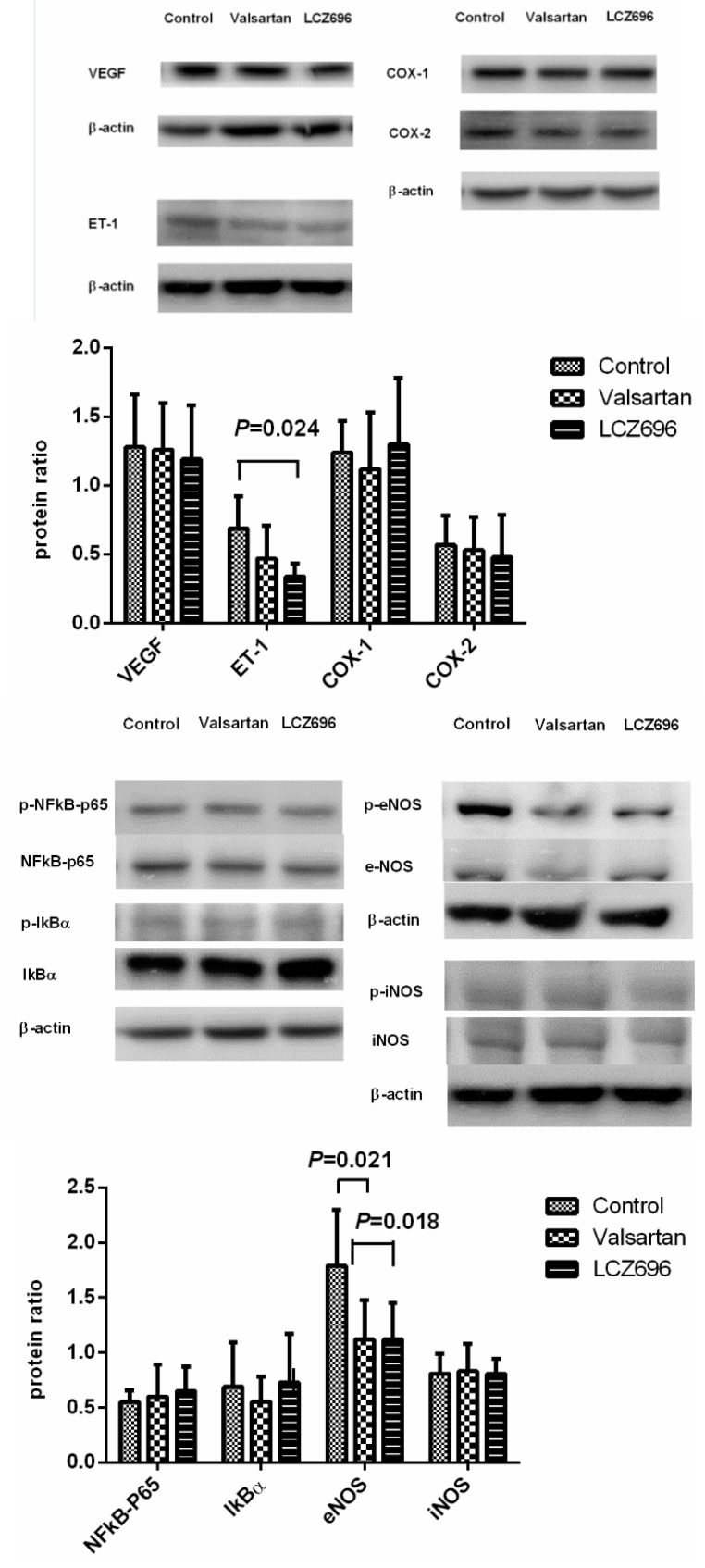
Hepatic protein expressions of control, valsartan-, LCZ696-treated PVL rats. The densitometric quantification and representative Western blot images are shown. The upper panel reveals that the endothelial-1 (ET-1) protein expression was significantly downregulated by LCZ696 compared to the control group. The vascular endothelial growth factor (VEGF), cyclooxygenase (COX)-1, and COX-2 protein expressions were not significantly different among control, valsartan-, and LCZ696-treated PVL rats. The lower panel indicates that the phosphorylated-endothelial nitric oxide synthase (eNOS) protein expressions were downregulated by valsartan and LCZ696 treatments. The phosphorylated-nuclear factor kappa B (NFκB) p65, phosphorylated-antinuclear factor of kappa light polypeptide gene enhancer in B-cells inhibitor alpha (IκBα), and phosphorylated-inducible nitric oxide synthase (iNOS) protein expressions were not affected by valsartan and LCZ696.

Figure 4 displays the Western blot of hepatic protein expressions after different treatments. LCZ696 significantly downregulated the hepatic protein expression of ET-1 (control vs. valsartan vs. LCZ696: 0.69 ± 0.23 vs. 0.47 ± 0.24 vs. 0.34 ± 0.09, LCZ696 vs. control: *P* < 0.05). Valsartan and LCZ696 also significantly downregulated the eNOS phosphorylation (control vs. valsartan vs. LCZ696: 1.79 ± 0.51 vs. 1.12 ± 0.36 vs. 1.12 ± 0.33, valsartan and LCZ696 vs. control: *P* < 0.05). The VEGF, COX-1, COX-2, phosphorylated-NFκB p65, phosphorylated-IκBα, and phosphorylated-iNOS protein expressions were not significantly influenced by valsartan and LCZ696 (VEGF/β-actin = 1.28 ± 0.38 vs. 1.26 ± 0.34 vs. 1.19 ± 0.39, COX-1/β-actin = 1.24 ± 0.23 vs. 1.12 ± 0.41 vs. 1.30 ± 0.48, COX-2/β-actin = 0.57 ± 0.21 vs. 0.53 ± 0.24 vs. 0.48 ± 0.31, phosphorylated-NFκB p65/NFκB p65 = 0.55 ± 0.11 vs. 0.60 ± 0.29 vs. 0.65 ± 0.22, phosphorylated-IκBα/IκBα = 0.69 ± 0.40 vs. 0.55 ± 0.23 vs. 0.73 ± 0.44, phosphorylated-iNOS/iNOS = 0.81 ± 0.18 vs. 0.83 ± 0.25 vs. 0.81 ± 0.13, all *P* > 0.05).

## 4. Discussion

The major findings of the present study were that (1) LCZ696 and valsartan reduced mean arterial pressure, which may be attributed to, at least partly, a decrease of systemic vascular resistance in sham-operated and portal hypertensive rats; (2) LCZ696, but not valsartan, reduced portal pressure and SMAR in portal hypertensive rats; (3) LCZ696 and valsartan improved hepatic biochemical data and reduced CD68-stained macrophage infiltrations in the liver. 

Our data showed that LCZ696 and valsartan significantly reduced MAP and SVR, which was in accordance to the previous studies in the hypertensive patients and experimental animal models [18,19]. Regarding portal hypertension, we found that LCZ696, but not valsartan, reduced PP and SMAR in portal hypertensive rats, implicating the superior portal hypotensive effects of LCZ696 compared to valsartan. Portal pressure was determined by PVf and intrahepatic resistance. Since the present study revealed that PVf was not significantly different among the LCZ696- and valsartan-treated and control groups, the portal hypotensive effect of LCZ696 might be related to the reduced intrahepatic resistance. The superiority of LCZ-696 compared to valsartan comes from its potent vasodilatory effects. LCZ696 can not only augment NPs but also influence other vasoactive agents [20]. Our data showed that LCZ696, but not valsartan, significantly downregulated the hepatic ET-1 protein expression. ET-1 is the main vasoconstrictor controlling intrahepatic resistance in portal hypertensive status, which accounts for, at least partly, the LCZ696-induced reduction of intrahepatic resistance. Indeed, the impacts of ARNI on ET-1 are different among various tissues. It is worth noting that neprilysin inhibition upregulated the ET-1 level, particularly in neprilysin-rich organs like the kidney [21]. However, another study showed that ARNI reduced the plasma ET-1 level in rats with pulmonary hypertension [22]. The various findings may be related to different research subjects and experimental settings. 

On the other hand, the current study found that LCZ696 and valsartan treatments downregulated the hepatic eNOS protein expressions in portal hypertensive rats. Since nitric oxide is a potent vasodilator, the paucity of intrahepatic nitric oxide synthase plays an important role by increasing intrahepatic resistance [1]. Initially, we postulated that LCZ696 treatments could upregulate eNOS protein to reduce intrahepatic resistance in PVL rats. However, a contradictory data showed LCZ696 and valsartan downregulated the hepatic eNOS protein expressions in portal hypertensive rats. We reviewed the literatures about LCZ696 and valsartan in the function of nitric oxide and vascular endothelium. In a study comparing the effect of LCZ696 to valsartan monotherapy, LCZ696 significantly ameliorated the impairment of acetylcholine-induced vascular relaxation, while it was not exerted by valsartan [23]. However, another study showed that LCZ696 was as effective as valsartan in improving the impaired endothelium-dependent hyperpolarization-mediated responses during hypertension, while no difference was observed in acetylcholine-induced, nitric oxide-mediated relaxations [24]. Indeed, the ARNI-induced hepatic ET-1 and eNOS downregulation in portal hypertensive rats has not been reported in the previous literature, and the ET-1 downregulation may play a dominant role in the LCZ696-induced portal hypotensive effect according to our data. This interesting finding suggests a complicated interplay of vasoactive substances during chronic LCZ696 treatment. 

Regarding other agents acting via modulating ET-1 and nitric oxide (NO) levels with potential portal hypotensive effects, bosentan is a nonselective ET-1 blocker and sildenafil is a phosphodiesterase which upregulates endothelial nitric oxide synthase. Bosentan has been documented to improve portal hypertension through amelioration of intrahepatic vasoconstriction [25]. On the other hand, low-dose sildenafil treatment for 1 week decreased intrahepatic resistance in rats with biliary cirrhosis, which was related to improve NO bioavailability [26]. However, a clinical study revealed that sildenafil did not influence hepatic venous pressure gradient in patients with cirrhosis [27]. Therefore, further large-scale clinical investigations may be required. 

In the present study, both LCZ696 and valsartan treatments improved liver biochemistry data, as evidenced by the decreased plasma levels of alanine aminotransferase. Although the H&E staining of the liver appeared similar among control and valsartan- and LCZ696-treated groups, the numbers of intrahepatic Cluster of Differentiation 68 (CD68)-stained macrophages were significantly decreased by LCZ696 and valsartan treatments. Although LCZ696 and valsartan did not influence the protein expressions of inflammatory markers as phosphorylated NF-κB, IκBα, and iNOS, the infiltration of macrophages in the liver is an important indicator of hepatic inflammation [28]. A previous report showed that ARNI ameliorated pulmonary inflammation and reduced the release of inflammatory mediators such as interleukin (IL)-6, IL-1β, and tumor necrosis factor (TNF)-α [29]. It has been noted that angiotensin II plays an active role in inflammation [30]. Furthermore, valsartan significantly inhibited adipose tissue macrophage infiltration and inflammation in rats treated by long-term high-fat diet [31]. In addition, valsartan has been shown to reduce CD68-stained cell adhesion to endothelial cell in spontaneously hypertensive rats [32]. Therefore, the antiinflammatory effect of ARNI and ARB might be contributed mainly by the blockade of angiotensin. 

RAAS blockade is commonly used to attenuate the progression of chronic kidney disease. Emerging data showed that LCZ696 is more effective than valsartan therapy alone in delaying the progression of kidney disease via antiinflammation, antioxidant, and antifibrosis effects [33]. In early stages of diabetic nephropathy, Habibi et al. [34] showed that LCZ696 was superior to valsartan in reducing proteinuria, renal ultrastructure, and tubular injury in a murine model. Furthermore, a recent study demonstrated that the renoprotection effect of LCZ696 was attributed by limiting podocyte injury [35]. In the present study, we found that LCZ696 and valsartan treatments did not affect the plasma level of creatinine, an indicator of renal injury. However, the comprehensive impact of LCZ696 on the renal angiotensin system of portal hypertension still awaits further clarification. 

Since ARNI elevates the NPs levels, some researchers raise concerns about the potential adverse effects of NPs in cirrhotic and portal hypertensive status [36,37]. It has been noted that the level of BNP is upregulated in cirrhotic patients and it is significantly related to complications, including esophageal varices and ascites [36]. In addition, the serum level of CNP concentration is also elevated in patients with chronic liver disease and is associated with unfavorable prognosis of cirrhotic patients [37]. However, in PVL rats, Jonas et al. [38] showed that the plasma ANP level was reduced in PVL rats compared to the sham rats. In addition, the ANP mRNA level was downregulated by 40% to 60% in the heart of PVL rats [38]. The discrepant results from portal hypertensive animals and patients disclose the complexity of vasoactive agents in portal hypertension. Patients with portal hypertension exhibit peripheral vasodilatation and inadequate intravascular effective volume, which elicit compensatory responses including enhanced sympathetic activity, RAAS activation, and elevated circulating vasopressin and ET-1 levels [39]. Therefore, the elevated NPs in patients with long-term portal hypertension might be merely the compensatory action to counteract hyperdynamic circulation. On the contrary, ARNI treatment started at the early stage may exert a therapeutic potential to block the development of portal hypertension. 

The dreadful complications of portal hypertension, including variceal bleeding and hepatic encephalopathy, are closely linked with portal-systemic collaterals. Emerging evidences show that BNP promotes vessel growth by increasing the number of endothelial progenitors and enhancing their functional properties, which may upregulate angiogenesis [40]. However, CNP has a contrary effect by counteracting vascular endothelial growth factor (VEGF) on angiogenesis [41]. CNP activation has been shown to inhibit key events of the angiogenic cascade, such as migration and proliferation of endothelial cells [42]. Regarding ANP, it has been documented to block VEGF signaling in vitro and to reduce VEGF-induced blood-retinal barrier leakage in vivo [43]. These divergent evidences show that NPs have pro- and antiangiogenesis properties in different tissues and situations. On the other hand, ARB also exerts antiangiogenesis activity. Valsartan markedly decreased capillary density in hamsters with cardiomyopathy by downregulating VEGF expression [44]. In rats with steatohepatitis, ARB significantly inhibited activated hepatic stellate cells and decreased the formation of new vessels both in vitro and in vivo [45]. Although our data showed that LCZ696 and valsartan treatments had a neutral effect on portal-systemic collateral formation and they did not affect intrahepatic VEGF protein expression in portal hypertensive rats, the impact of ARNI treatment on cirrhotic status awaits further exploration. 

In conclusion, LCZ696 and valsartan reduce mean arterial pressure, which is related to a reduction of systemic vascular resistance. Besides, LCZ696, but not valsartan, exerts the portal hypotensive effect and a decrease of intrahepatic resistance, which might be contributed by hepatic ET-1 downregulation. Both LCZ696 and valsartan improve liver biochemistry data and reduce hepatic CD68-positve staining macrophages. The effects of LCZ696 on portal hypertensive animals suggest its therapeutic potential in this field.

## Figures and Tables

**Table 1:** Body weight and hemodynamic parameters of sham-operated rats.

	Control (*n* = 6)	Valsartan (*n* = 5)	LCZ696 (*n* = 5)
BW (g)	356 ± 26	367 ± 8	367 ± 26
MAP (mmHg)	145 ± 11	121 ± 19 *	104 ± 15 *
PP (mmHg)	10.4 ± 0.6	8.8 ± 2.2	9.5 ± 1.1
HR (beats/min)	352 ± 23	359 ± 41	353 ± 24
PVf (mL/min/100 g)	9.8 ± 0.9	9.8 ± 4.1	8.1 ± 1.6
SMAf (mL/min/100 g)	6.2 ± 1.1	5.4 ± 1.0	6.0 ± 1.5
SMAR(mmHg/mL/min/100 g)	22.6 ± 5.9	21.4 ± 6.0	16.4 ± 3.8
SVR (mmHg/mL/min/100 g)	3.9 ± 0.4	3.2 ± 0.6 ^#^	2.7 ± 0.5 *
CI (mL/min/100 g)	37.6 ± 4.4	38.8 ± 6.0	39.2 ± 7.9

BW, body weight; MAP, mean arterial pressure; PP, portal pressure; HR, heart rate; PVf, portal venous flow; SMAf, superior mesentery arterial flow; SMAR, superior mesentery arterial resistance; SVR, systemic vascular resistance; CI, cardiac index; * *P* < 0.05 compared to the control group, ^#^ *P* = 0.054 compared to the control group.

**Table 2:** Body weight and hemodynamic parameters of portal hypertensive rats.

	Control (*n* = 5)	Valsartan (*n* = 7)	LCZ696 (*n* = 7)
BW (g)	304 ± 20	285 ± 16	287 ± 30
MAP (mmHg)	126 ± 5	103 ± 16 *	90 ± 12 *
PP (mmHg)	15.4 ± 1.6	14.0 ± 2.3	12.0 ± 2.0 *
HR (beats/min)	323 ± 26	349 ± 27	325 ± 47
PVf (mL/min/100 g)	3.4 ± 1.4	4.8 ± 2.1	4.4 ± 1.9
SMAf (mL/min/100 g)	6.0 ± 0.6	7.9 ± 2.7	7.3 ± 1.8
SMAR (mmHg/mL/min/100 g)	18.6 ± 1.8	12.9 ± 5.7	11.3 ± 3.6 *
SVR (mmHg/mL/min/100 g)	3.9 ± 0.4	2.7 ± 1.1 *	2.3 ± 0.5 *
CI (mL/min/100 g)	32.5 ± 3.6	41.2 ± 11.3	39.6 ± 5.7

BW: body weight; MAP: mean arterial pressure; PP: portal pressure; HR: heart rate; PVf: portal venous flow; SMAf: superior mesentery arterial flow; SMAR: superior mesentery arterial resistance; SVR: systemic vascular resistance; CI: cardiac index; * *P* < 0.05 compared to the control group.
